# Antibody Array Revealed PRL-3 Affects Protein Phosphorylation and Cytokine Secretion

**DOI:** 10.1371/journal.pone.0169665

**Published:** 2017-01-09

**Authors:** Yongyong Yang, Shenyi Lian, Lin Meng, Like Qu, Chengchao Shou

**Affiliations:** 1 Key Laboratory of Carcinogenesis and Translational Research (Ministry of Education), Department of Biochemistry and Molecular Biology, Peking University Cancer Hospital & Institute, Beijing, China; 2 Key Laboratory of Carcinogenesis and Translational Research (Ministry of Education), Department of pathology, Peking University Cancer Hospital & Institute, Beijing, China; Murdoch University, AUSTRALIA

## Abstract

Phosphatase of regenerating liver 3 (PRL-3) promotes cancer metastasis and progression via increasing cell motility and invasiveness, however the mechanism is still not fully understood. Previous reports showed that PRL-3 increases the phosphorylation of many important proteins and suspected that PRL-3-enhanced protein phosphorylation may be due to its regulation on cytokines. To investigate PRL-3’s impact on protein phosphorylation and cytokine secretion, we performed antibody arrays against protein phosphorylation and cytokines separately. The data showed that PRL-3 could enhance tyrosine phosphorylation and serine/threonine phosphorylation of diverse signaling proteins. Meanwhile, PRL-3 could affect the secretion of a subset of cytokines. Furthermore, we discovered the PRL-3-increased IL-1α secretion was regulated by NF-κB and Jak2-Stat3 pathways and inhibiting IL-1α could reduce PRL-3-enhanced cell migration. Therefore, our result indicated that PRL-3 promotes protein phosphorylation by acting as an ‘activator kinase’ and consequently regulates cytokine secretion.

## Introduction

The human genome contains about 500 genes encoding protein kinases, and the majority of them are serine/threonine (S/T) kinases and about 90 are tyrosine (Y) kinases [1]. Reversible tyrosine phosphorylation is regulated by the balanced action of protein tyrosine kinases (PTKs) and protein tyrosine phosphatases (PTPs). Aberrant tyrosine phosphorylation resulting from dysregulated PTP activity has been implicated in the progression of various diseases, including cancer, diabetes, and rheumatoid arthritis [2].

The Phosphatase of Regenerating Liver (PRL) phosphatases are a distinct sub-family of prenylated protein-tyrosine phosphatases consisting of three members (PRL-1, 2, and 3) that share over 75% of amino acid sequence identity [3]. PRL-3 was initially found to be associated with colon cancer metastasis [4]. Subsequent studies revealed that PRL-3 was abundant in many cancer cell lines and metastatic lesions, including gastric cancer [5], malignant melanoma cancer [6], ovarian cancer [7], breast cancer [8], colonic cancer [9], glioma [10], multiple myeloma [11], hepatocellular carcinoma [12], intrahepatic cholangio-carcinoma [13], esophageal squamous cell carcinoma [14], lung carcinoma [15], chronic and acute myeloid leukemia [16, 17], and salivary adenoid cystic carcinoma [18]. High level of PRL-3 is associated with a poor prognoses and PRL-3 has been proposed as a potential biomarker for evaluating tumor aggressiveness [19]. Evidence showed PRL-3 could promote EMT via decreasing PTEN expression and activating PI3K-AKT signaling [20], regulating cadherin-related signaling pathway and cadherin directly [21], and enhancing KCNN4 channels [22]. PRL-3 was found to promote the motility, invasion, and metastasis through PRL-3-integrin β1-ERK1/2 and MMP2 signaling [23, 24], or through a NF-κB-HIF-1α-miR-210 axis [25]. PRL-3 was also shown to promote cell invasion and proliferation by Csk down-regulation and Src activation [26, 27]. In addition, PRL-3 regulates cell migration through ADP-ribosylation factor 1 (Arf1)-activity-dependent stimulation of integrin α5 recycling [28]. A recent study showed that PRL-3 could activate mTOR by increasing PI3K/Akt-mediated activation of Rheb-GTP via TSC2 suppression [29]. Besides, PRL-3 was shown to be an important cell-cycle regulator and a target of p53 [30], while PRL-3 could down-regulate p53 by enhancing expression of PIRH2, which is a negative regulator of p53 [31].

To date, only few phosphorylated proteins were reported as PRL-3’s substrates, i.e., Ezrin [32], Elongation factor 2 (EF-2) [33], Keratin 8 (KRT8) [34], Integrin β1 [24], Stathmin [35] and Nucleolin [36]. However, various studies showed that PRL-3 could activate diverse signaling pathways by promoting protein phosphorylation [26, 29, 37, 38]. It has been reported that PRL-3 expressing cells exhibited a pronounced increase in protein tyrosine phosphorylation and intracellular activation of the extensive signaling network [26, 37, 38], which was speculated to be governed by extracellular ligand-activated transmembrane secreted factors [37, 38], however, this speculation remains to be validated.

Antibody microarray has been widely used for comprehensive proteomic analysis in various cancers and other diseases [39]. Here in our study, for purpose of further investigating the impact of PRL-3 on protein phosphorylation, including tyrosine phosphorylation and serine/threonine phosphorylation, we conducted phosphorylation antibody array. Our result confirmed that PRL-3 increased both tyrosine phosphorylation and serine/threonine phosphorylation of proteins related to many crucial signaling pathways. In the mean time, cytokine antibody array was performed, which showed that PRL-3 could increase the secretion of several cytokines. Additionally, we discovered that PRL-3-increased IL-1α secretion was affected by NF-κB and Jak2-STAT3 signaling pathways and IL-1α was essential for PRL-3 enhanced cell migration. We suggest that PRL-3 increased protein phosphorylation could participate in the regulation of cytokine secretion, which may contribute to cancer metastasis and progression and other biological processes induced by the aberrant expression of PRL-3.

## Materials and Methods

### Ethics statement

The study using human tissue samples was approved by the Biomedical Ethical Committee of Peking University Cancer Hospital & Institute. Colorectal cancer tissue samples were obtained from the Tissue Bank of Peking University Cancer Hospital. These samples were surgically dissected, pathologically verified, and stored in liquid nitrogen frozen. Written informed consent was obtained from all patients.

### Cell lines, reagents and antibodies

The human colon cancer cell lines HCT116 and LoVo were obtained from ATCC (Manassas, VA, USA). HCT116 and LoVo cells stably expressing PRL-3 and control cells (NC) were previously established [40]. NF-κB inhibitor Bay 11–7082 (S2913) and Jak2 inhibitor AG490 (S1143) were obtained from Selleckchem (Houston, TX, USA). IL-6 (TP723240) was obtained from OriGene Technologies (Rockville, MD, USA). Recombinant human IL-1RN (ag1277) and anti-GAPDH (10494-1-AP) were from Proteintech Group (Chicago, IL, USA). Anti-Myc tag (AB103) was from TianGen Biotech (Beijing, China). Mouse anti-PRL-3 monoclonal antibody was generated by immunizing mice with KLH-conjugated full-length human PRL-3. Anti-phosphoserine/threonine (612548) was from BD Bioscience (San Jose, CA, USA). Anti-p65 (sc-372) was from Santa Cruz Biotechnology (Dallas, TX, USA). Anti-phosphotyrosine (9411), anti-pS536-p65 (3033), anti-stat3 (9139), anti-pS705-stat3 (9145), anti-pS727-stat3 (9134), anti-Jak2 (3230), anti-pY1007/1008-Jak2 (3776), anti-Erk (9102), anti-pT202/Y204-Erk1/2 (9106), anti-EGFR (4267), anti-pY1045-EGFR (2237), anti-pY1068-EGFR (3777), anti-pY527-Src (2105), anti-pT180/Y182-p38 (4511), anti-pT581-MSK1 (9595), and anti-pT334-MAPKAPK2 (3007) were purchased from Cell signaling (Danvers, MA, USA).

### Cell culture

Cells were normally grown in RPMI-1640 (Invitrogen, Carlsbad, CA, USA), supplemented with 10% bovine serum, 100 U/ml of streptomycin and penicillin in a Thermo cell incubator (5% CO_2_). For collecting culture supernatants, cells were harvested at about 80% density and washed by PBS twice gently, and then cultured in RPMI-1640 without FBS for 24 hours.

### Western blot

Cells were harvested from culture dish with 2×SDS loading buffer. Colorectal cancer tissues were homogenized in RIPA buffer containing protease inhibitors. Polyacrylamide gel electrophoresis (SDS-PAGE) and Western blotting were performed as previously described [40].

### Phospho-protein antibody array and cytokine antibody array

Cells was collected and delivered to Wanyen Biotechnologies Inc (Shanghai, China) in liquid nitrogen. Protein was extracted and was examined for phosphorylation using an antibody array (Full Moon BioSystems; catalog PEX100) containing well characterized site-specific antibodies against 582 phosphoproteins and their 452 unphosphorylated counterparts. Antibody array slides were scanned by GenePix 4000B (Axon Instruments) and data were calculated by GenePix Pro 6.0 (Axon Instruments). The phospho ratio of the specific phospho-site was calculated as below:

Phospho ratio = (phospho _PRL-3_/unphospho _PRL-3_)/ (phospho _NC_/unphospho _NC_).

Significantly changed phosphoproteins (p < 0.05) over 1.5-fold up- or 0.5-fold downregulated were included. The entire data of phosphoprotein array was shown in S1 Table.

For the secreted cytokine analysis, cells were grown in RPMI-1640 without FBS for 24 hours and the supernatants were delivered to RayBiotech Inc (Guangzhou, China) in liquid nitrogen. Culture supernatants were added to antibody arrays against 1000 unique cytokines (RayBiotech; catalog AAH-BLG-1000) and processed according to the manufacturer’s protocol. The array slides were also scanned by GenePix 4000B (Axon Instruments) and data were calculated by GenePix Pro 6.0 (Axon Instruments). Significantly changed cytokines (p < 0.05) over 1.5-fold up- or 0.5-fold down-regulated when the signal value in HCT116-NC or HCT116-PRL-3 was greater than 200 were included. The data of cytokine array was shown in S2 Table.

### Bioinformatics analysis

The translation of Gene ID of significantly changed phosphoproteins was performed using DAVID Bioinformatics Resources (version 6.7, http://david.abcc.ncifcrf.gov/), as well as the GO (Gene Ontology) analysis of the differential phosphoproteins and cytokines based on the biological process, protein class, molecular function and cellular component. We further used DAVID to investigate expressively regulated pathways and p-values were corrected for multiple testing by using a B-H (Benjamini-Hochberg) procedure. The data generated by DAVID was shown in S3 Table. Heat maps were generated with Heatmap illustrator (Heml, version 1.0). The protein-protein interaction analysis of significantly regulated kinases was performed using STRING 10 (http://string-db.org/) and further visualized by Cytoscape 3.2.1.

### Quantitative Real-time PCR (qRT-PCR)

Total RNA was extracted from cells with Trizol reagent (Invitrogen), and then 1 μg of RNA was used to synthesize cDNA with GoScriptTM Reverse Transcription system (A5001, Promega). qRT-PCR was performed using a StepOne Real-time PCR system (Applied Biosystems) and SYBR Green PCR master mix reagents (TOYOBO). Expression data were normalized to that of GAPDH. Primers used are listed in S4 Table.

### Measurement of the secreted cytokines

After culture supernatants were collected as described above, GDF-15, IL-1α and Neuropeptide Y (NPY) in supernatant was assayed by using the enzyme immunoassay kits according to the procedures described by the manufacturers. IL-1α (DLA50) and GDF-15 (DGD150) were purchased from R&D Systems (Minneapolis, MN, USA), while NPY (E-EL-H1893c) was from Elabscience (Wuhan, Hubei, China).

### Knockdown of IL-1α in HCT116-NC/PRL-3 cells

The IL-1α and negative control small interfering RNAs (siRNAs) were purchased from GenePharma (Shanghai, China). The sequences of each siRNA are as follows: si-IL-1α-1#, sense: 5’-GUUCCUCCAUUGAUCAUCUTT-3’, antisense: 5’-AGAUGAUCAAUGGAGGAACTT-3’; si-IL-1α-2#, sense: 5’-CUGAAGAAGAGACGGUUGATT-3’, antisense: 5’-UCAACCGUCUCUUCUUCAGTT -3’; si-negative control (NC): sense: 5’-UUCUCCGAACGUGUCACGUTT-3’, antisense: 5’-ACGUGACACGUUCGGAGAATT-3’. Cells were plated at a density of 20 × 10^4^ cells/well in six-well plates. 50 nM IL-1α or NC siRNA was added to the cells by using Oligofectamine from GenePharma (Shanghai, China) according to the manufacturer’s protocol. Fresh medium was replaced 48 hours later, and then the cells were collected and used for migration assays or for preparation of total RNA extraction.

### Cell migration assay

200 μl re-suspended cells containing 5 ng IL-1RN or after knockdown of IL-1α were seeded on the upper chamber of each transwell (Becton Dickinson, San Jose, CA, USA) at a density of 2×10^5^/ml, with 800 μl medium containing 10% FBS added to the lower chamber. Cells were allowed to migrate at 37°C for 48 hours. The transwells were fixed in methanol and stained in 0.1% crystal violet solution for 30 min at room temperature. Cells in the upper chamber were removed with a cotton wool. The polycarbonate membrane was then removed and sealed on slide by resin, and the cells that penetrated to the lower side of the membrane were counted in six randomly selected fields under a ×100 or ×200 microscope.

### Statistical analysis

Each experiment was done independently at least three times with similar results. The data were analyzed by using Prism 5 (GraphPad Software, San Diego, CA, USA). Results expressed as the mean ± SD. Significant differences were assessed with the Student’s t test and p < 0.05 was considered significant.

## Results

### Universally raised phosphorylation by PRL-3 overexpression

**Fig 1:**
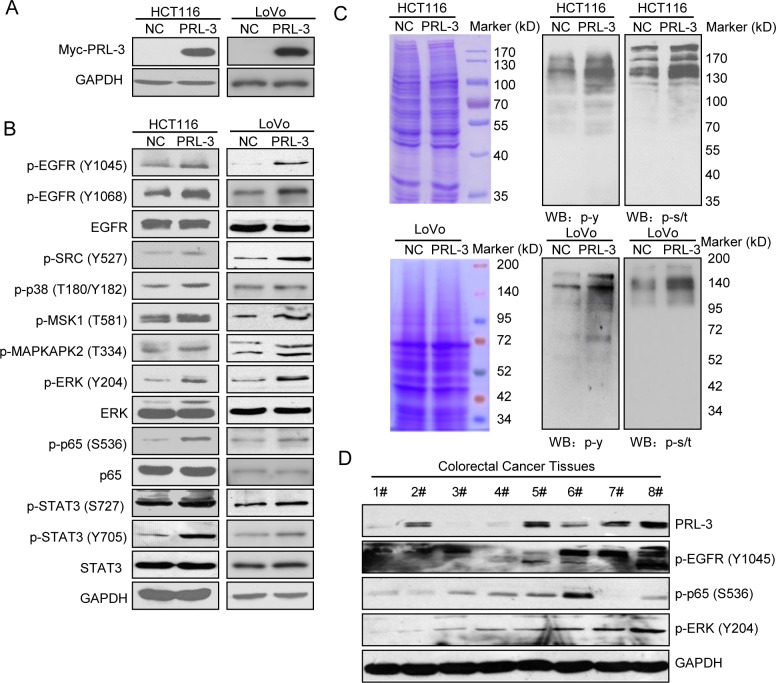
Overexpression of PRL-3 widely increased protein phosphorylation. (A) Confirmation of PRL-3 overexpression in HCT116 and LoVo cells. (B) Several phosphoproteins increased by PRL-3 in HCT116 and LoVo cells. (C) PRL-3’s wide impact on the whole protein phosphorylation, including tyrosine phosphorylation and serine/threonine phosphorylation in HCT116 and LoVo cells. (D) PRL-3’s impacts on phosphorylations of EGFR, p65, and ERK in eight colorectal cancer tissues.

We constructed PRL-3 overexpressing cells in HCT116 and LoVo cells by stably transfecting pcDNA3.1-myc-NC/PRL-3 as described earlier [24, 40] and confirmed the over-expression of PRL-3 by Western Blot (Fig 1A). Previous reports demonstrated enhanced tyrosine phosphorylation in PRL-3 overexpressing cells [26, 37, 38], and various studies showed that PRL-3 could activate several signaling pathways by regulating protein phosphorylation [23, 29, 38, 41]. Here we confirmed phosphorylation of several important proteins in our system, including EGFR, SRC, p38, MSK1, MAPKAPK2, ERK, p65 and STAT3 (Fig 1B). Then we investigated PRL-3’s impact on protein phosphorylation using pan-specific anti-phosphorylation antibodies. The result showed that PRL-3 universally increased tyrosine phosphorylation (Fig 1C), which was consistent with previous reports [26, 37, 38]. The serine/threonine phosphorylation was also broadly increased in PRL-3 overexpressing cells (Fig 1C). The total protein content was measured by Coomassie brilliant blue staining, which showed almost equal loading quantity of protein samples. The result suggested that PRL-3-increased protein phosphorylation is not solely limited to tyrosine phosphorylation or serine/threonine phosphorylation. To substantiate data from cultured cells overexpressing PRL-3, we also detected protein phosphorylation in eight freshly isolated colorectal cancer tissues. As shown in Fig 1D, profiles of PRL-3 expression was distinct in different samples, while phosphorylations of EGFR, p65 and ERK were largely correlated with PRL-3 levels, suggesting that PRL-3 also plays a role in stimulating protein phosphorylation in vivo.

### Phosphoproteomic analysis of the differentially regulated phosphoproteins

**Fig 2:**
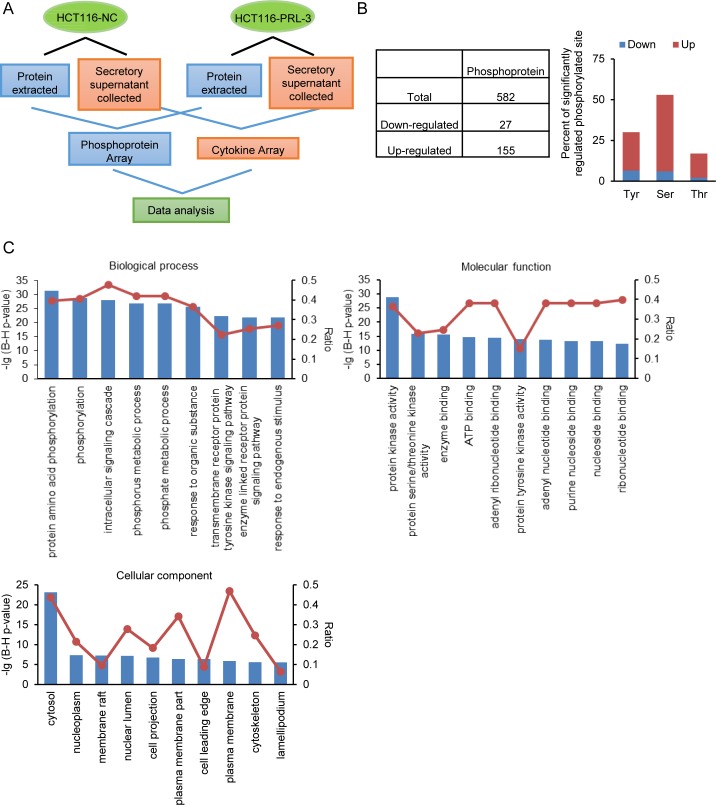
Go analysis of significantly regulated phosphoproteins. (A) Workflow of the analysis of PRL-3’s impact on protein phosphorylation and cytokine secretion in HCT116 cells by antibody array. (B) PRL-3 induced phosphoproteome profile. Total number of phosphoproteins, and percent of phosphorylated Ser (S), Thr (T), and Tyr (Y) in the total significantly regulated phosphoproteins. (C) Functional characterization of the significantly regulated phosphoproteins, including biological processes, cellular components and molecular functions.

Given the importance of protein phosphorylation in cellular signal transduction [42], we performed phosphorylation antibody array analysis in HCT116-NC/PRL-3 cells as the workflow described in Fig 2A. We identified that there were 182 significantly changed protein phosphorylations corresponding to 135 proteins when compared the PRL-3 overexpressing cells with the control cells. Among the significantly changed protein phosphorylations, 155 protein phosphorylations were upregulated, and 27 protein phosphorylations were downregulated (Fig 2B). The result further showed that PRL-3 increases not only tyrosine phosphorylation, but also serine/threonine phosphorylation (Fig 2B), which was in agreement with results of Western Blotting experiment (Fig 1B). The data of top 20 significantly upregulated phosphoproteins was listed in Table 1.

**Table 1:** The top 20 phosphoproteins experiencing changes in phosphorylation in the PRL-3 overexpressing cells.

phosphoprotein	Official Full Name (Swiss Prot; Gene ID)	ratio	function of phosphor-site ^a^
STAT1 (P-Ser727)	signal transducer and activator of transcription 1(P42224;6772)	3.03	apoptosis; cell growth; transcription; molecular association; sumoylation; enzymatic activity;
CDK7 (P-Thr170)	cyclin-dependent kinase 7(P50613;1022)	3.05	transcription; enzymatic activity;
P70S6K (P-Ser418)	ribosomal protein S6 kinase B1 (P23443;6198)	3.05	enzymatic activity;
EGFR (P-Tyr1092)	epidermal growth factor receptor (P00533;1956)	3.12	apoptosis; cell adhesion; cell growth; enzymatic activity; intracellular localization; molecular association; phosphorylation; protein conformation; protein degradation; receptor internalization; ubiquitination;
Caspase 9 (P-Ser144)	caspase 9(P55211; 842)	3.16	apoptosis; enzymatic activity;
E2F1 (P-Thr433)	E2F transcription factor 1(Q01094;1869)	3.24	cell differentiation; intracellular localization; protein degradation;
IKK-b (P-Tyr188)	inhibitor of kappa light polypeptide gene enhancer in B-cells, kinase beta (O14920;3551)	3.37	transcription; enzymatic activity;
JAK2 (P-Tyr221)	Janus kinase 2(O60674;3717)	3.41	enzymatic activity;
BCR (P-Tyr177)	breakpoint cluster region(P11274;613)	3.42	apoptosis; carcinogenesis; cell growth; cell motility; molecular association; phosphorylation;
Pyk2 (P-Tyr402)	protein tyrosine kinase 2 beta (Q14289;2185)	3.43	cell cycle regulation; cell motility; enzymatic activity; intracellular localization; molecular association; phosphorylation;
FosB (P-Ser27)	FBJ murine osteosarcoma viral oncogene homolog B(P53539;2354)	3.60	protein stability; nuclear localization;
FAK (P-Tyr407)	protein tyrosine kinase 2 (Q05397;5747)	3.63	cell spreading, migration, and survival;
Androgen Receptor (P-Ser650)	androgen receptor(P10275;367)	3.66	transcription;
HSP 90-beta (P-Ser226)	heat shock protein 90kDa alpha family class B member 2, pseudogene(P08238;391634)	3.80	apoptosis; molecular association;
Src (P-Ser75)	SRC proto-oncogene, non-receptor tyrosine kinase(P12931;6714)	3.84	cell growth; cytoskeletal reorganization;
IKK-a/b (P-Ser180/181)	conserved helix-loop-helix ubiquitous kinase/inhibitor of kappa light polypeptide gene enhancer in B-cells, kinase beta(O15111/O14920;1147/3551)	3.86	cell motility; transcription; protein stabilization;
AKT1 (P-Tyr474)	v-akt murine thymoma viral oncogene homolog 1 (P31749;207)	3.89	enzymatic activity;
PKC alpha (P-Thr638)	protein kinase C alpha(P17252;5578)	4.17	intracellular localization;
PKD1/PKC mu (P-Tyr463)	polycystin 1, transient receptor potential channel interacting(Q15139; 5310)	4.24	apoptosis; cell differentiation; transcription;
Pyk2 (P-Tyr580)	protein tyrosine kinase 2 beta (Q14289;2185)	4.29	enzymatic activity;

^*a*^Function of phosphor-site referenced PhosphoSitePlus (http://www.phosphosite.org).

To gain insight into the biological processes, molecular function and the subcellular locations of these differentially regulated proteins, we identified associated Go terms using DAVID. As shown in Fig 2C, the top ten biological processes included regulation of phosphorylation, phosphorus and phosphate metabolic, response to organic substance and endogenous stimulus, and transmembrane receptor protein tyrosine kinase signaling pathway, indicating many significantly changed phosphoproteins were associated with regulation of phosphorylation. Besides, many of these phosphoproteins act as protein kinases and ATP, DNA, or RNA binding proteins and they are distributed in the cytosol, nucleoplasm, plasma membrane, and cytoskeleton. As protein phosphorylation normally influences protein kinase activity, thus the fact that the phosphorylation levels of 44 protein kinases were markedly changed suggested PRL-3 might regulate a spectrum of protein phosphorylations by modulating several upstream kinases’ phosphorylation status (S3 Table). Furthermore, the protein-protein interaction between these kinases indicated that IKBKB, LYN, PRKCA, JAK2, MTOR and SRC may play key roles as upstream kinases when PRL-3 regulates phosphoproteins (S1 Fig).

**Fig 3:**
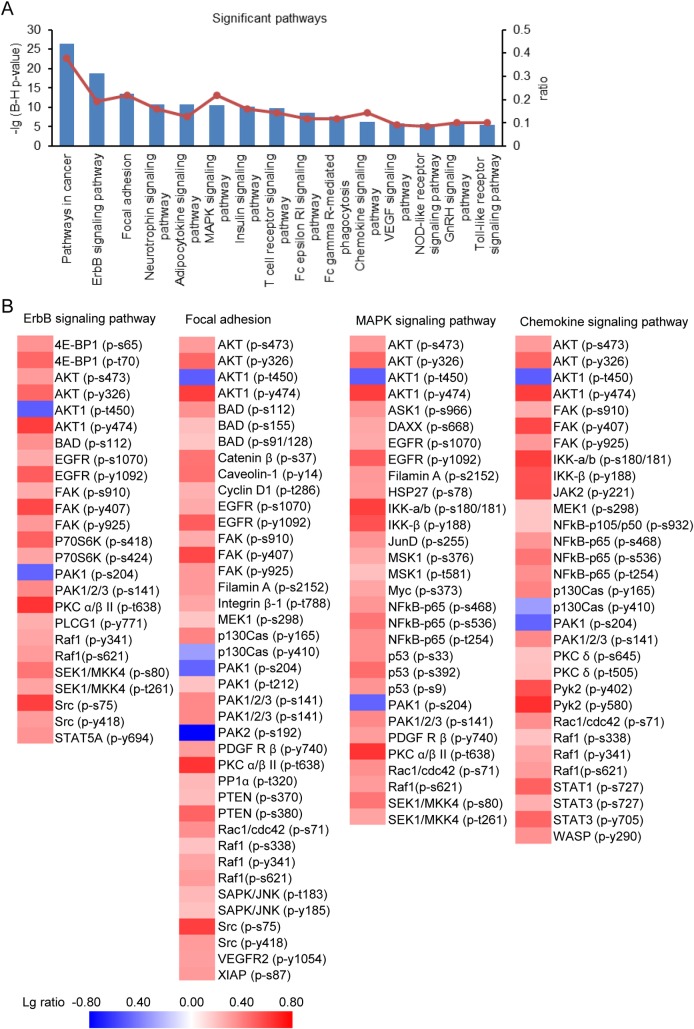
Identification of significant pathways regulated by PRL-3. (A) Significant KEGG pathways identified by DAVID when PRL-3 was overexpressed in HCT116 cells. The left-side ordinate values are -lg (Benjamini-Hochberg p-value), while the right-side ordinate values are the ratios of phosphorylated proteins in the total significantly regulated phosphoproteins. (B) Heatmaps of several pathways to show the phosphoproteins regulated by PRL-3, including ErbB signaling pathway, Focal adhesion, MAPK signaling pathway and Chemokine signaling pathway. The red color represents the phosphorylation of the protein was increased in PRL-3 overexpressing cells, while the blue color represents the phosphorylation of the protein was decreased in PRL-3 overexpressing cells.

We further used DAVID to examine the regulated phosphoproteins’ participation in signal transduction pathways and the top 13 signal pathways predicted to be expressively represented from our data set are shown in Fig 3A. Deregulated phosphorylation has been implicated in cancer and some phosphoproteins could be cancer markers useful for diagnostics and therapeutics [2, 43]. Some significantly changed phosphoproteins (34.19%) were involved in pathway in cancer. PRL-3 could induce EGFR activation through down-regulation of protein tyrosine phosphatase 1B (PTP1B) [38]. Here our data further confirmed that PRL-3 increased the phosphorylation levels of EGFR at tyrosine 1092 and serine 1070 and its downstream signaling components and 15.38% of the significantly regulated phosphoproteins were associated with ErbB signaling pathway (Fig 3A and 3B). PRL-3 could down-regulate the tyrosine-phosphorylation level of integrin β1 and enhance phosphorylation level of Erk1/2 [24]. It was also demonstrated that PRL-3 inhibits the transcriptional regulation of integrin α2 signaling or modulates the recycling of integrin α5 [28]. As reported above, our analysis here showed that PRL-3 was a participant in focal adhesion, likely via modulating the phosphorylation of various focal adhesion components, such as integrin, PAK and FAK (Fig 3B). In addition, there was a report showed that VEGF promotes the transcription of PRL-3 [43], meanwhile PRL-3 has an undefined role in modulating VEGF signaling [41]. Here our data described the impact of PRL-3 on VEGF signaling pathway by up-regulating the phosphorylation of VEGFR2 at tyrosine 1054 and 1214, as well as its key downstream targets, such as PLCG1, PKC α/β/δ/ε/θ, PTK2 and SRC (S1 Table). PRL-3-activated mTOR signaling pathway was also reflected in our data (S1 Table), which was consistent previous study [29]. We also showed some PRL-3 regulated phosphoproteins in several signaling pathways, such as MAPK signaling pathway and Chemokine signaling pathway (Fig 3B). Up to now, most of molecular mechanisms potentially affected by PRL-3 could be confirmed by our study, suggesting our data was validated and comprehensive. More importantly, our analysis also revealed that PRL-3 could play important roles in Neurotrophin signaling pathway, Adipocytokine signaling pathway, MAPK signaling pathway, Insulin signaling pathway, T cell receptor signaling pathway, Fc epsilon RI signaling pathway, Fc gamma R-mediated phagocytosis, Chemokine signaling pathway, NOD-like receptor signaling pathway, GnRH signaling pathway and Toll-like receptor signaling pathway, suggesting PRL-3 may take part in the regulation of nervous system, energy metabolism, immune system and other physiological activities through regulation of protein phosphorylations (Fig 3A). As a tyrosine phosphatase, there is limited information about PRL-3’s substrates. Our result discovered that PRL-3 reduced the phosphorylation levels of 27 proteins (S1 Table), and some of them could be the substrates of PRL-3 when it functions as a phosphatase, which deserves more study to verify.

It is worth to note that the analysis of phosphoprotein array also revealed that mutation diminishing the phosphatase activity (PRL-3/C104S) could increase protein phosphorylation as the wild type PRL-3 did (S2A and S2B Fig), suggesting the impact of PRL-3 on protein phosphorylation was not definitely dependent on its phosphatase function, which was inconsistent with previous report that showed there was almost no difference in protein tyrosine phosphorylation between the vector control and PRL3/C104S cells [37]. The fact that PRL-3 overexpressing cells were constructed in different cells may give rise to the difference, as they constructed PRL-3 overexpressing cells by using HEK293 cells, while we used HCT116 cells.

### PRL-3’s impact on cytokine secretion

**Fig 4:**
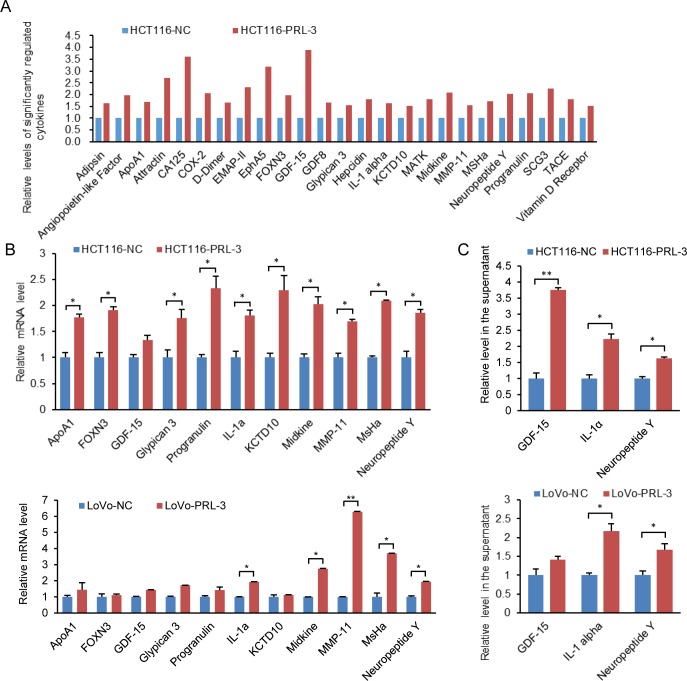
Significantly regulated cytokines by PRL-3. (A) Significantly regulated cytokines were all increased in PRL-3 overexpressing HCT116 cells. (B) The mRNA levels of several regulated cytokines were increased in PRL-3 overexpressing HCT116 and LoVo cells. (C) Relative levels of GDF-15, IL-1α and NPY in the supernatants of PRL-3 overexpressing HCT116 and LoVo cells and corresponding control cells. The values are the mean and standard deviation, *p <0.05, **p <0.01; n = 3.

As indicated in phosphoproteins-associated signaling pathway (Fig 3A), PRL-3 is involved in the regulation of adipocytokine and chemokine, suggesting PRL-3 could regulate cytokine secretion. Here we detected the impact of PRL-3 on cytokine secretion by performing antibody array in the supernatant of HCT116-PRL-3 cells and the control cells as described in the workflow (Fig 2A). We identified 25 significantly changed cytokines and these 25 cytokines were all up-regulated when PRL-3 was overexpressed (Fig 4A). We then studied PRL-3’s impact on the mRNA levels of some differentially changed cytokines, and most of them were increased in PRL-3 expressing cells (Fig 4B), indicating that PRL-3-regulated cytokine production is associated with transcriptional regulation. We further confirmed the secretion of GDF-15, IL-1α and NPY using ELISA kits and they were all increased in the supernatants of PRL-3 overexpressing cells (Fig 4C). We also investigated the impact of the mutant PRL-3 on cytokine secretion using antibody array and the result showed that mutation at the phosphatase motif of PRL-3 could significantly regulated the secretion of several cytokines, and all the regulated cytokines in PRL-3M cells were increased (S2C Fig).

### PRL-3 enhanced IL-1α production is associated with NF-κB and Jak2-Stat signal pathways

**Fig 5:**
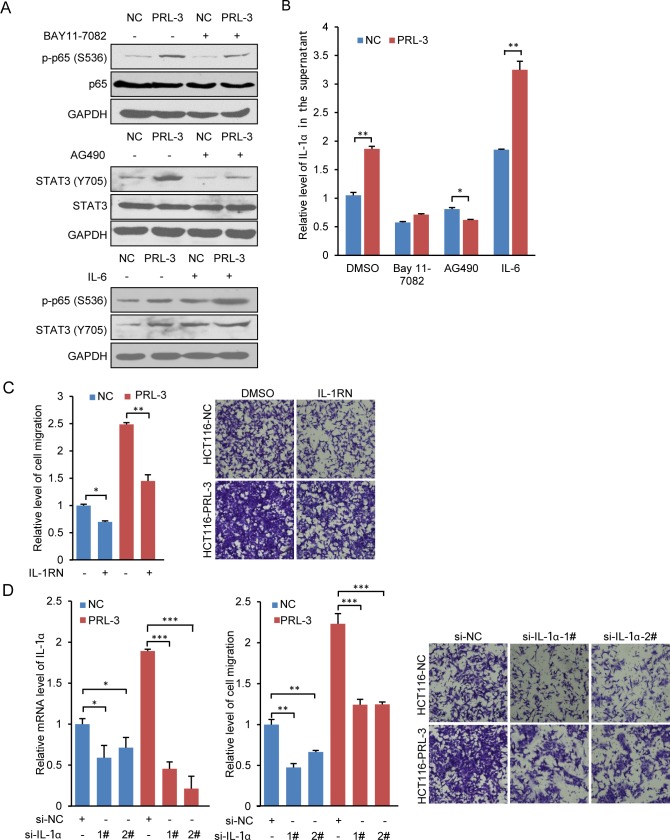
IL-1α participates in PRL-3 induced cell migration. (A) and (B) IL-1α was regulated by PRL-3 through NF-κB and Jak2-STAT3 signaling pathways. (A) The treatment of HCT116 cells with 10 μM BAY11-7082 for 24 hours reduced PRL-3-increased p-p65 (S536), and the treatment of HCT116 cells with 2 μM AG490 for 24 hours reduced PRL-3-increased p-STAT3 (Y705) while the treatment of HCT116 cells with 100 ng/mL IL-6 for 24 hours increased p-p65 (S536) and p-STAT3 (Y705). (B) The treatment of HCT116 cells with 10 μM BAY11-7082 or 2 μM AG490 for 24 hours reduced PRL-3-increased IL-1α, and the treatment of HCT116 cells with 100 ng/mL IL-6 for 24 hours enhanced PRL-3-induced IL-1α secretion. (C) The treatment of HCT116 cells with 5 ng/mL IL-1RN for 48 hours decreased PRL-3-induced HCT116 cell migration. (D) Inhibition of IL-1α by siRNA interference decreased PRL-3-induced HCT116 cell migration. The values are the mean and standard deviation, * p < 0.05, ** p < 0.01, *** p < 0.001; n = 3.

IL-1 family is crucial for innate inflammatory and immune responses and can be induced by a wide variety of factors [44]. Recently, a report showed BMP-6 stimulates tumor-associated macrophages to produce IL-1α through a crosstalk between Smad1 and NF-kB1 [45]. In the present study, we found PRL-3 increased the production of IL-1α in HCT116 and LoVo cells (Fig 4). Because of the central role of NF-κB and STAT3 in modulating the production of cytokine, we treated HCT116-PRL-3 cells and the control cells with NF-κB and Jak2-STAT3 inhibitors and then the IL-1α levels in culture supernatants was measured by an ELISA kit. The inhibiting treatment decreased the phosphorylation of NF-κB and Jak2-STAT3 (Fig 5A) and reduced IL-1α secretion (Fig 5B), indicating that PRL-3 increased IL-1α secretion may be dependent on NF-κB and Jak2-STAT3 pathways. We next treated cells with IL-6, a cytokine known to activate both NF-κB and STAT3 [46, 47]. PRL-3’s stimulatory effects on p65 and STAT3 were further potentiated by IL-6 (Fig 5A), and PRL-3-induced IL-1α secretion was also enhanced (Fig 5B). Therefore, PRL-3 activated NF-κB and Jak2-STAT3 could affect the production of IL-1α, and IL-1α in turn could bind to membrane receptor, leading to the indirect activation of NF-κB and Jak2-STAT3 [48–51], thus probably forming a regulation loop and promoting PRL-3’s function in cell signaling. It has been reported that IL-1β could promote cell migration through activating MAPK and NF-κB [52], so we wondered whether IL-1α was associated with PRL-3-promoted cell migration. IL-1RN inhibits the activities of IL-1α and IL-1β by binding to receptor IL1R1. Here we found that blocking the IL-1 signaling by 5 ng IL-1RN significantly decreased cell migration of the control cells and PRL-3-overexpressing cells (Fig 5C). Moreover, knockdown of IL-1α by siRNA also markedly reduced cell migration of the control cells and PRL-3-overexpressing cells (Fig 5D). Thus, IL-1α was associated with PRL-3-promoted cell migration.

## Discussion

Our data showed that PRL-3 could affect multiple signaling pathways by enhancing tyrosine phosphorylation and serine/threonine phosphorylation in cultured cells and colorectal cancer tissues. Besides, PRL-3 could have impact on the secretion of several cytokines. Additionally, we found that PRL-3 promotes the secretion of IL-1α through activating NF-κB and Jak2-Stat3 pathways and the inhibition of IL-1α signaling could decrease PRL-3-enhanced cell migration.

It has been accepted that PRL-3 is associated with cancer metastasis and progression, however, the detailed function of PRL-3 remains unclear. As a phosphatase, there have been limited substrates of PRL-3 discovered so far. Contrary to the molecular nature of PRL-3 itself, PRL-3 could activate Src, Rho-family GTPases, PI3K-AKT, MAPK, STAT3/5, NF-κB, EGFR, and mTOR, and their downstream molecules by increasing phosphorylation levels [20, 26, 29, 38, 40, 53], thus functioning as a global ‘kinase activator’. Since protein kinases and phosphatases regulate multiple physiologies processes, PRL-3’s effect on protein tyrosine phosphorylation was investigated by mass spectrometry-based approach and PRL-3 was shown to significantly increase protein tyrosine phosphorylation [37]. In our study, we studied PRL-3’s impact on protein phosphorylation by conducting phosphorylation antibody array analysis. Consistent with previous report [26, 37, 38], our study demonstrated that there were increased protein tyrosine phosphorylation in PRL-3 overexpressing cells, and furthermore, we also discovered that there were much more up-regulated serine/threonine phosphorylations than the down-regulated serine/threonine phosphorylations when PRL-3 was overexpressed. Our result suggested PRL-3’s impact on protein phosphorylation was not limited to tyrosine phosphorylation or serine/threonine phosphorylation. More importantly, we found that PRL-3 was likely involved in Neurotrophin signaling pathway, Adipocytokine signaling pathway, T cell receptor signaling pathway, Fc epsilon RI signaling pathway, Fc gamma R-mediated phagocytosis, and chemokine signaling pathway, therefore expanding PRL-3’s possible effects on physiological processes, which deserve more research to identify. Besides, the mutation at the phosphatase motif didn’t obviously affect PRL-3’s impact on protein phosphorylation, suggesting PRL-3’s phosphatase activity could be dispensable for PRL-3 increased protein phosphorylation.

However, there is still no clear clue to explain how PRL-3 functions as an upstream activator of kinase. We hypothesize that PRL-3 may increase protein phosphorylation through activating several important kinases, and among them, IKBKB, LYN, PRKCA, JAK2, MTOR and SRC may be the crucial ‘relay stations’ and ‘amplifiers’ for PRL-3’s wide impact on protein phosphorylation.

Previous report conjectured that PRL-3 might regulate protein phosphorylation via extracellular factors [37]. In our study, the cytokine antibody array analysis uncovered 25 significantly changed cytokines after PRL-3 overexpression, and it is interesting that the secretion level of them were all increased by PRL-3 overexpression, suggesting PRL-3 could promote the secretion of a subset of cytokines. Additionally, we also tested the impact of PRL-3 mutation on cytokines by antibody array, and the data showed that most of cytokines significantly changed by the PRL-3 mutant were raised as in wild-type PRL-3 overexpressing cells. Unfortunately, we didn’t obtain direct proof that PRL-3 increases global phosphorylation through improving some specific extracellular factors, at least for these 25 changed cytokines. However, some identified cytokines may play roles in PRL-3-regulated signaling pathways. For example, increased adipocyte are positively correlated with the generation of leptin, which is a crucial regulator of energy intake and metabolic rate mainly through working at hypothalamic nuclei [54]. Leptin exerts its anorectic effects by modulating the levels of neuropeptides such as Neuropeptide Y (NPY), Agouti-related peptide (AgRP), and α-melanocyte-stimulating hormone (α-MSH) and this leptin action is through the JAK kinase, STAT3 phosphorylation, and nuclear transcriptional effect [54, 55]. In our study, we showed PRL-3 was associated with adipocytokine signaling pathway by bioinformatics analysis, and PRL-3 activated JAK kinase and increases STAT3 phosphorylation. Meanwhile, the detection of cytokines showed PRL-3 could increase NPY secretion, suggesting NPY increased by PRL-3 maybe participates in PRL-3 regulated adipocytokine signaling pathway.

Due to the important role of IL-1 in innate inflammatory and immune responses, we studied the mechanism of PRL-3 regulating the secretion of IL-1α, and found that PRL-3-increased IL-1α was influenced by NF-κB and Jak2-Stat3. Both NF-κB and Stat3 could function as transcriptional factors when they regulate cytokines’ expression such as IL-1β, and they share the same binding site on the promoter of IL-1β [56], so we propose that they might regulate IL-1α mainly through transcriptional regulation as previous report described [45]. Blocking IL-1 signaling by IL-1RN inhibited cell migration of both the control cell and PRL-3overexpressing cells, suggesting either IL-1α or IL-1β was associated with PRL-3 increased cell migration. Knockdown of IL-1α further demonstrated IL-1α could affect cell migration and contribute to PRL-3-increased cell migration, which may be also dependent on NF-κB and Jak2-Stat3.

Taken together, our study provided detailed and valuable data about PRL-3’s impact on protein phosphorylation and cytokine secretion, and revealed that PRL-3 is associated with many important signaling pathways, providing the clue of investigating the mechanism of PRL-3’s function in tumor progression and other physiological processes.

## Supporting Information

S1 Fig The protein-protein interactions of significantly changed kinases in PRL-3 overexpressing HCT116 cells. (TIF)

S2 Fig Analysis of PRL-3M’s impact on protein phosphorylation and cytokine secretion in HCT116 cells. (TIF)

S1 Table The entire data of phosphoprotein array. (XLSX)

S2 Table The entire data of cytokine array. (XLSX)

S3 Table The data generated by DAVID Bioinformatics Resources. (XLSX)

S4 Table Primers for RT-PCR. (XLSX)

S5 Table Data of significantly changed kinases. (XLSX)
