# Supplementary figures and images for: Antibody Array Revealed PRL-3 Affects Protein Phosphorylation and Cytokine Secretion
Source: PLoS One. 2017 Jan 9;12(1):e0169665. doi: 10.1371/journal.pone.0169665 (PMC5222497; doi:10.1371/journal.pone.0169665)

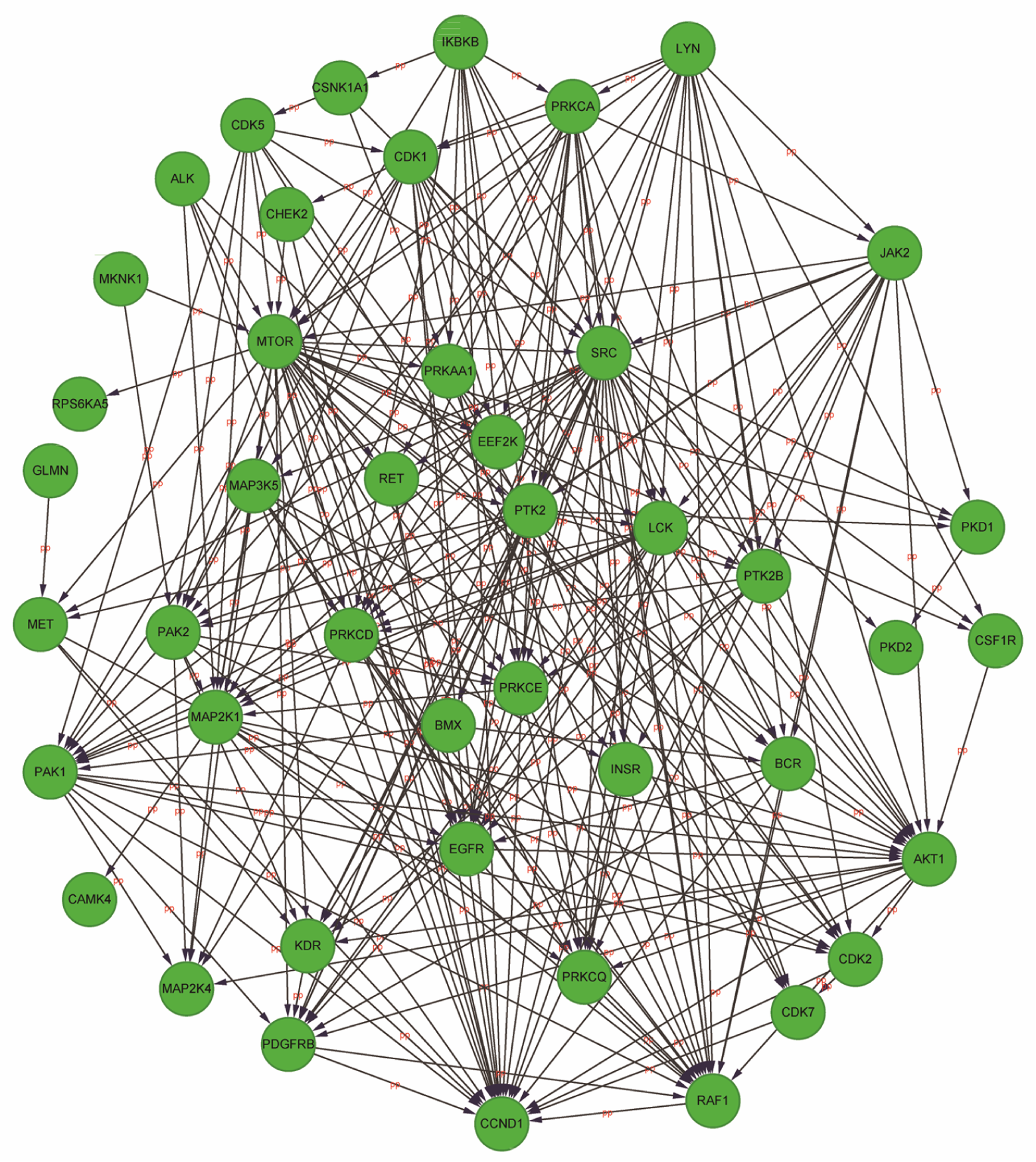

Supplement: S1 Fig — (TIF) [file pone.0169665.s001.tif]

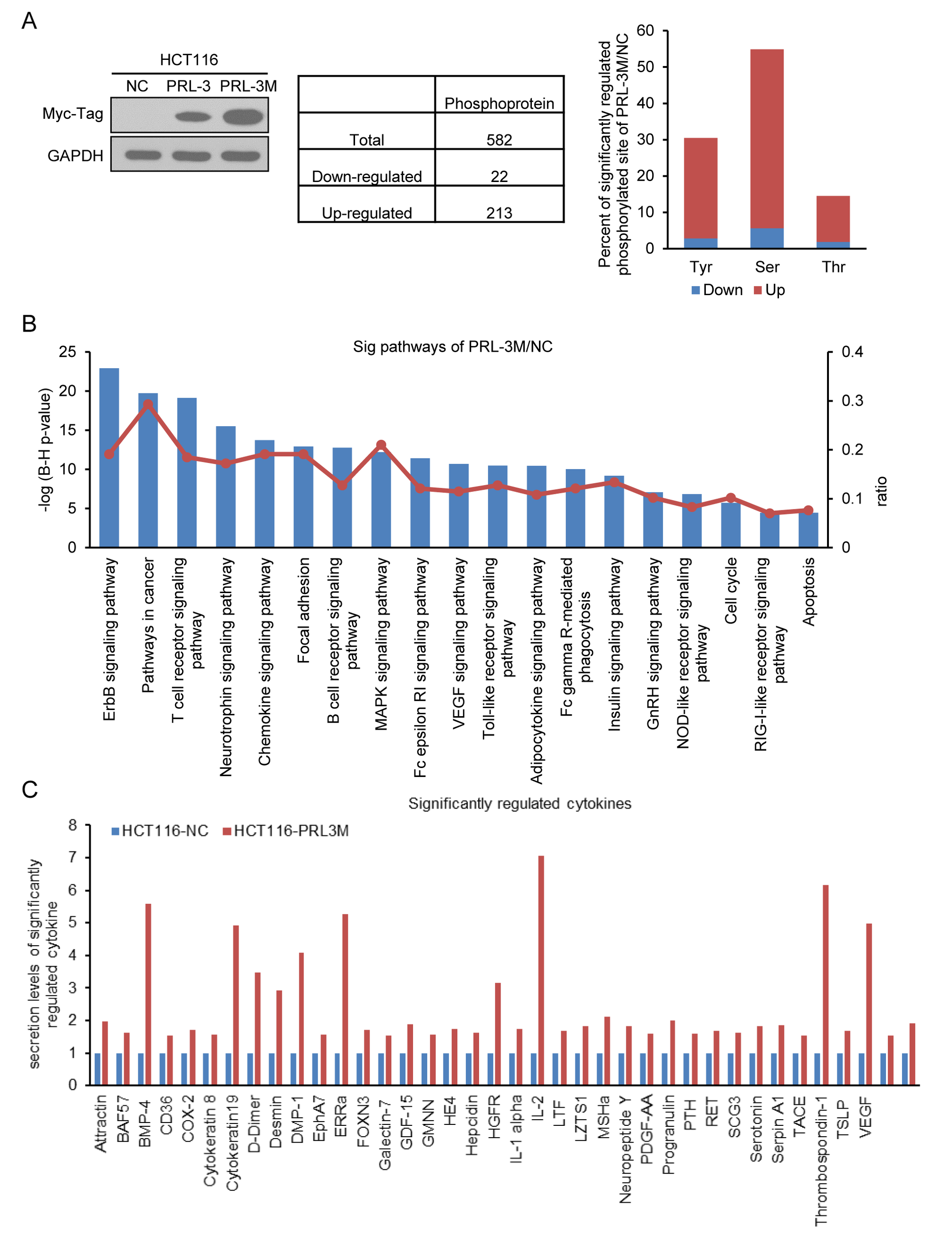

Supplement: S2 Fig — (TIF) [file pone.0169665.s002.tif]
